# Plasma expression of long non-coding RNA *GAS5* and its prognostic significance in newly diagnosed multiple myeloma

**DOI:** 10.3389/fonc.2026.1791554

**Published:** 2026-04-22

**Authors:** Anica Pravdic Divac, Natasa Tosic, Zorica Cvetkovic, Marko Radulovic, Teodora Karan-Djurasevic, Aleksandra Tomic, Irena Marjanovic, Natasa Stanisavljevic, Branka Zukic, Olivera Markovic

**Affiliations:** 1 Department of Hematology, University Clinical-Hospital center “Bezanijska kosa”, Belgrade, Serbia; 2 Institute of Molecular Genetics and Genetic Engineering, University of Belgrade, Belgrade, Serbia; 3 Department of Hematology, University Clinical-Hospital center Zemun, Belgrade, Serbia; 4 Faculty of Medicine, University of Belgrade, Belgrade, Serbia; 5 Clinic for Neurosurgery, University Clinical Center of Serbia, Belgrade, Serbia

**Keywords:** GAS5 expression, long-non coding RNA, multiple myeloma, plasma, prognosis

## Abstract

**Introduction:**

Long non-coding RNAs (lncRNAs) participate in pathogenesis, progression and therapy resistance in multiple myeloma (MM). Growth Arrest-Specific 5 (*GAS5*) is a lncRNA with a role in proliferation, invasion and metastasis in solid tumors, but prognostic significance in MM is unknown so far. Aim: The purpose of this study was to examine the expression pattern of *GAS5* lncRNA in plasma samples of MM patients and to evaluate its clinical and prognostic significance.

**Methods and material:**

73 newly diagnosed patients with MM and 16 healthy volunteers were recruited from Clinical Hospital Centre (CHC) “Bežanijska Kosa” and CHC “Zemun” in the period from July 2020 to December 2024. Blood samples were collected before starting therapy. Demographic, laboratory and clinical parameters, prognostic score, overall response rate, progression free survival (PFS) and overall survival (OS) were analyzed. Relative quantification analysis of the *GAS5* expression level was performed by RQ-PCR methodology with the *GAPDH* gene as endogenous control and by using the comparative ddCt method with control samples as a calibrator.

**Results:**

No significant difference was found between plasma expression *GAS5* in MM patients (median 0.833, range 0.013-3.678) and *GAS5* expression in healthy control samples (median 1, range 0.49-2.668) (p=0.116). In multivariate analysis with PFS as the dependent variable, higher *GAS5* expression and advanced age were associated with shorter time to progression, while higher R2-ISS score, advanced age and occurrence of relapse/progression of disease were independent prognostic factors for survival in our group of patients.

**Conclusion:**

This is the first study showing prognostic potential of circulating lncRNA *GAS5* in the MM patients. Patients with MM had a slightly lower *GAS5* expression compared to control samples, this was, nevertheless, without statistical significance. In patients with higher GAS5 expression disease progression occurred earlier compared to patients with lower expression. To establish *GAS5* as a robust biomarker in MM, future studies should focus on larger patient cohorts to validate its expression within the bone marrow niche and its specific association with high-risk ‘double-hit’ cytogenetic aberrations and glucocorticoid resistance.

## 1 Introduction

Multiple myeloma (MM) represents the most prevalent hematological malignancy following non-Hodgkin lymphoma and leukemia and recently the median overall survival of these patients is more than 10 years (1, 2). The prognosis for patients with MM has improved significantly in recent decades. Longer survival in MM is largely the result of the introduction of new drug combinations. The recommended treatment for these patients is the use of a combination of proteasome inhibitors, immunomodulatory drugs, monoclonal antibodies, following with autologous hematopoietic stem cell transplantation (ASCT). In relapse or refractory disease, according to the latest guidelines, the use of CAR-T cells and bispecific antibodies is suggested. The use of glucocorticoids has been the backbone of MM treatment for decades and its use is recommended at every stage of treatment, from treatment of smoldering MM to symptomatic active MM, further through successive relapses toward the most aggressive terminal stage, plasma cell leukemia (3). However, a small number of patients experience an unfavorable outcome and the length of survival is several months from the moment of diagnosis. The reason for the poor outcome in MM is largely the result of patient characteristics (older age, presence of comorbidities), aggressive biology of MM (complex genetic heterogeneity) and the development of therapeutic resistance (4, 5).

Much attention has been directed towards the investigation of epigenetic changes, in the last few years, in order to elucidate the clinical and genetic heterogeneity in MM. Several studies have shown that epigenetic changes in MM are potentially key factors in varying prognosis and therapeutic response in these patients (6). Epigenetic changes represent alterations in gene expression that do not lead to structural changes in the gene sequence. Numerous studies have shown that epigenetic alterations (changes in DNA methylation, histone modification, expression of micro RNAs and long non-coding RNAs) play an important role in pathogenesis, clinical and therapeutic outcome in patients with MM (7–9).

Long non-coding RNAs (lncRNAs) represent a class of RNAs whose transcript length exceeds 200 nucleotides, and they produce non-functional protein. However, lncRNAs are involved in key cellular processes such as differentiation, proliferation and cell death (10). LncRNAs may have an oncogenic or tumor-suppressive effect in MM, thus influencing the therapeutic response and the outcome of disease. In addition, some lncRNAs have been shown to be potential prognostic biomarkers (11).

Growth arrest-specific transcript 5 (*GAS5)* is a type of lncRNA originally identified as a key regulator of cell cycle and development. Previous studies have confirmed that *GAS5* overexpression is observed in growth-arrested cells (12). The *GAS5* gene is localized on chromosome 1q25 and its transcript is approximately 650 nucleotides long (13). Transcription of the *GAS5* gene results in ten small nucleolar RNAs (snoRNAs) of the C/D box type, in addition to two mature lncRNA isoforms (*GAS5a* and *GAS5b*) (14). For the most part, *GAS5* performs its function through small snoRNAs (15). *GAS5* achieves its biological role through three key molecular mechanisms. Primarily, *GAS5* could act as a signaling molecule, for example, in colorectal cancer, DNA damage promotes the expression of *GAS5*, which activates the p53 signaling pathway and induces apoptosis (16). Secondly, *GAS5* may be molecular “sponge” that blocks binding sites intended for other molecules (proteins or RNAs). Similarly, this mechanism was confirmed in interaction between *GAS5* and glucocorticoid receptors (GR) where *GAS5* inhibits anti-inflammatory and apoptotic effects of glucocorticoids (17). Finally, *GAS5* serves as a transport mediator for transcription factors, thereby indirectly regulating the transcription of specific target genes. It was previously reported that *GAS5* affects the connection between the transcription factor 1 E2F and promoter of the cyclin-dependent kinase inhibitor 1B gene, through modifying its transcription (18).

To date, the molecular mechanisms of *GAS5* have been extensively investigated in cancer pathogenesis (19). Traditionally, *GAS5* has been recognized as a tumor-suppressor lncRNA and is frequently downregulated across numerous malignancies (20–23). Furthermore, *GAS5* expression has been shown as a prognostic factor in several solid tumors. Reduced *GAS5* expression is associated with advanced disease stage, the development of metastases, therapeutic resistance and poorer prognosis (24–28). Recent evidence has indicated that *GAS5* overexpression may also have function as an oncogene in specific solid tumors by modulating expression miR423-3p in hepatocellular carcinoma and miR1287 in cholangiocarcinoma. Furthermore, even in the absence of a defined molecular pathway, elevated *GAS5* levels have been clinically associated with poor outcomes in kidney clear cell carcinoma (29–32). Similarly, investigations into the function of *GAS*5 in hematological malignancies have contradictory findings. While downregulation of *GAS5* has been linked to a poorer prognosis in acute myeloid leukemia (33) and diffuse large B-cell lymphoma (34), its overexpression is associated with a poor prognosis in myelofibrosis (35) and poor therapy response in childhood B-acute lymphoblast leukemia (36). These findings suggest that the role of *GAS5* is highly context-dependent, acting either as a tumor suppressor or an oncogene depending on the specific hematological malignancy and its unique microenvironment.

In MM, *GAS5* expression has been rarely investigated, and to the best of our knowledge there are only two previously published studies offering conflicting results (37, 38) Therefore, in our study, we aimed at analyzing the expression pattern of *GAS*5 in the plasma in MM patients and also, at evaluating association between *GAS*5 expression level and clinical parameters of the MM patients, and at examining its potential influence on prognosis of disease (early therapeutic stratification, progression-free survival (PFS) and overall survival (OS)).

## 2 Material and methods

### 2.1 Patients and methods

A total of 73 newly diagnosed patients with MM and 16 healthy control samples were prospectively included from the Departments of Hematology at Clinical Hospital Centre (CHC) “Bežanijska Kosa” and CHC “Zemun” (Belgrade, Serbia) in the period from July 2020 to December 2024. The control group consisted of 16 hospital-based healthy individuals without any apparent disease. They were clinically tested for the absence of MM and any other malignant tumors. The diagnosis and follow-up of MM were conducted according to the current 2021 EHA-ESMO guidelines (3). Additionally, relevant cytogenetic abnormalities (t (4;14), del(17p), t (14;16), t (14;20), amp1q and del 1p) were detected using interphase Fluorescence *in situ* Hybridization. Patients were stratified and characterized based on established prognostic scoring systems, specifically the International Staging System (ISS) (39), the Revised International Staging System (R-ISS) (40), and the R2-ISS (41). Double hit MM is evaluated as the presence 2 of any high-risk cytogenetic abnormalities (42).

The therapy administered to the patients was in accordance with the available treatment modalities at specified times and in agreement with the national guidelines established by the Serbian Myeloma Working Group (43). The majority of patients (n=61; 84%) received a bortezomib-based regimen. Five patients (7%) were treated with a thalidomide-based regimen. Almost all patients received a three-drug combination therapy, except for six patients (8%) who were treated with a two-drug regimen (bortezomib-dexamethasone or melphalan-prednisone combinations). ASCT was performed only on 11 patients (15%). The primary reasons for not performing ASCT in the remaining cohort were often related to advanced age, a high comorbidity index, progressive disease, or technical constraints. Response to treatment was defined based on the 2016 Revised IMWG Criteria (44). Regular follow-up was maintained through either telephone contact or scheduled clinic visits. The last follow-up date for all participants was September 2025.

PFS was defined as time from the date of diagnosis until the date of disease progression or death from any cause. OS was measured from the date of diagnosis to the date of death from any cause (45). For patients who were lost during follow-up, the OS date was set as the date of their last clinic visit when they were known to be alive.

With the provision of informed consent, peripheral blood samples from patients with MM and healthy controls were collected. The study was performed in accordance with the guidelines of the Declaration of Helsinki Principles and Good Clinical Practice. Sample collection was made with full informed consent of the patients and ethics committee of the relevant healthcare institutions that approved this study.

### 2.2 RNA isolation, reverse transcription, and RT-qPCR

Blood samples were collected in EDTA-containing tubes and processed within 2 h for plasma collection in order to prevent hemolysis. Cell and cellular component free plasma samples were obtained using two-step centrifugation protocol. First, blood samples were centrifuged for 10 min at 1900 g, at 4 °C, and then a second centrifugation step was performed to eliminate any cell debris (10 min at 16–000 g, at 4 °C). Supernatant was collected in aliquots of 500 μl and stored at –80 °C until RNA extraction and thawed only once. Blood samples with hemolysis were excluded.

Total RNA was isolated from 500 μl of plasma using miRNeasy Serum/Plasma Kit and QIAvac24 Plussystem (Qiagen, Germany) according to manufacturer’s instructions. The final concentration and purity of obtained RNA was measured using Nano Drop (Shimatzu, Japan). Reverse transcriptase (RT) reaction was performed using RevertAid Reverse Transcriptase (Thermo Fisher Scientific, Waltham, MA, USA). The relative expression of *GAS5* in plasma cells was detected by reverse transcription−quantitative PCR (RT−qPCR) done on 7900 HT Fast Real-Time PCR System (Thermo Fisher Scientific, Waltham, MA, USA). We performed PCR using TaqMan chemistry, TaqMan Universal Master Mix II and TaqMan Gene Expression Assay for *GAS5* (Hs03464472_m1) and for GAPDH (Hs99999905_m1) as an endogenous control (Thermo Fisher Scientific, Waltham, MA, USA). GAPDH was chosen as an endogenous control based on previous reports showing that its expression in plasma is stable and not affected by age, sex or pathology (46, 47). All PCR reactions were performed in triplicate, and the thermal profile was 95 °C for 10 min, followed by 50 cycles of 95 °C for 15 s, and 60 °C for 1 min. Relative expression of the *GAS5* was calculated using ddCt method with the healthy control as a calibrator.

## 3 Statistical analyses

All statistical analyses were performed using SPSS version 24.0. Normally distributed data are presented as mean ± SD, whereas non-normally distributed data are presented as median and interquartile ranges (IQR). Categorical variables are presented as counts and percentages. Comparisons were analyzed using the independent-samples t-test, chi-square test, and Mann–Whitney U test. Correlations between GAS5 expression levels and clinical or laboratory parameters were assessed using Spearman’s rank correlation coefficient. Patients were stratified into high-expression and low-expression groups using the median value of *GAS5* expression as the cut-off point. Progression-free survival (PFS) and overall survival (OS) of *GAS5^high^* and *GAS5^low^* group were analyzed using Kaplan–Meier curves and the log-rank test. Cox regression was used for both univariable and multivariable analyses. Variables included in the multivariable model were selected based on a univariable significance threshold of 0.1 and LASSO regularization. All tests were two-sided, and *P* < 0.05 was considered statistically significant.

## 4 Results

### 4.1 Demographic and clinical characteristics of patients

The mean age of patients was 69, 4 ± 9, 5 years, and almost a quarter of patients are older than 75 years (n=20, 27%)). Thirty-nine (53%) were males, and 34(47%) were females. According to the type of MM, distribution was as follows: 46 IgG myeloma patients (63%), 15 IgA patients (20.5%), and 12 light chain patients (16.5%). Advanced clinical stage III (Durie&Salmon) had 60 patients (82%), 8 (11%) were in clinical stage II, and 5 (7%) in clinical stage I of disease. The distribution of patients according ISS and R-ISS stage 1/2/3 is as follows: 9 (12%)/17 (23%)/47 (65%) and 6 (8%)/46 (64%)/20 (28%), respectively. Patients were stratified according to R2-ISS in low (n=6, 8%), low-intermediate (n=17, 24%), high-intermediate (n=44, 61%) and high-risk group (n=5, 7%). The double hit MM was registered by the following distribution: 3 (4%) patients presented with the co-occurrence of del (17p) and amp (1q), and 6 (8%) patients had a combination of t (4, 14) and amp (1q). Therapy response was achieved in 60 (82%) patients. Relapse or progression of disease was registered in 28 (38%) patients. Median of follow-up was 29 months. Overall mortality rate was 36% (n=26). Characteristics of the entire group of MM patients as well as the patients with high and low *GAS5* expression were shown in **Table 1**.

**Table 1:** Characteristics of MM patients (n=73) with high(n=37) and low(n=36) *GAS5* expression.

Characteristics	Total	*GAS5* ^high^	*GAS5* ^low^	p
Mean age ± SD, years	69.4 ± 9.5	69.7 ± 10.5	69 ± 8.5	0.783
Sex, M/F, n (%)	39 (53)/34 (47)	16 (41)/21 (62)	23 (59)/13 (38)	0.070
Comorbidity, yes/no, n (%)	61 (84)/12 (16)	29 (48)/8 (67)	32 (52)/4 (33)	0.226
Laboratory results				
Hb, mean ± SD, g/l	96.9 ± 20.7	99.6 ± 22.7	94.2 ± 18.3	0.240
Plt, mean ± SD, x10^9^/l	213 ± 85.4	217.8 ± 8.5	208.1 ± 88.2	0.737
Ca, mean ± SD, mmol/l	2.4 ± 0.4	2.4 ± 0.3	2.5 ± 0.5	0.060
LDH, median (range), ULN	315 (83-718)	332 (83-662)	282 (129-718)	0.168
SCR, median (range), µmol/l	107 (37.1-1146)	104.3 (51-660)	114.9 (37.1-1146)	0.235
Alb, mean ± SD, g/l	34.2 ± 7.9	33.9 ± 8.9	34.4 ± 6.7	0.120
TP, mean ± SD, g/l	86.2 ± 21.7	83.7 ± 21.8	88.9 ± 21.7	0.945
β2M, median (range), ng/ml	6.7 (1.5-41.7)	6.5 (1.5-28)	7.75 (2.26-41.7)	0.500
Immunoglobulin subtype, n (%)				0.420
IgG	46 (63)	21 (46)	25 (54)	
IgA	15 (21)	8 (53)	7 (47)	
Light chain	12 (16)	8 (67)	4 (33)	
Clinical stage (Durie-Salmon), n (%)				0.911
1	5 (7)	3 (60)	2 (40)	
2	8 (11)	4 (50)	4 (50)	
3	60 (82)	30 (50)	30 (50)	
Cytogenetic abnormality, yes/no, n (%)				
del17p	6 (8)/67 (92)	4 (67)/33 (49)	2 (33)/34 (51)	0.414
t (4, 14)	11 (15)/62 (85)	7 (64)/30 (48)	4 (36)/32 (52)	0.351
t (14, 16)	1 (1)/72 (99)	0 (0)/37 (51)	1 (100)/35 (49)	0.307
amp1q21	19 (26)/54 (74)	13 (68)/24 (44)	6 (32)/30 (56)	0.072
Double hit	9 (12)/64 (88)	8 (89)/29 (45)	1 (11)/35 (55)	0.014
Prognostic scores				
ISS, 1, 2 vs. 3, n (%)	26 (36)/47 (64)	15 (58)/22 (47)	11 (42)/25 (53)	0.373
R-ISS, 1, 2 vs.3, n (%)	53 (74)/19 (26)	25 (47)/11 (58)	28 (53)/8 (42)	0.422
R2-ISS, 1, 2 vs.3, 4, n (%)	23 (32)/49 (68)	12 (52)/24 (49)	11 (48)/25 (51)	0.800
Treatment response, CR, VGPR, PR vs. SD, PD, n (%)	54 (78)/15 (22)	23 (43)/10 (67)	31 (57)/5 (33)	0.099
Relapse or progression, yes/no, n (%)	28 (42)/39 (58)	17 (61)/15 (38)	11 (39)/24 (62)	0.072
Outcome, alive/dead, n (%)	47 (64)/26 (36)	20 (43)/17 (65)	27 (57)/9 (35)	0.062

Dichotomization was performed using the median expression value. Hb, hemoglobin; Plt, platelets; LDH, lactate dehydrogenases; SCR, serum creatinine; Alb, albumins; TP, total proteins; CRPC, reactive protein; β2M, β2-microglobulin; ISS, International Staging System; R-ISS, revised ISS; R2-ISS, Second RISS; CR, complete response; VGPR, very good partial response; PR, partial response; SD, stable disease; PD, progression of disease.

### 4.2 Expression level of plasma *GAS5* and clinicopathological characteristics

**Figure 1:**
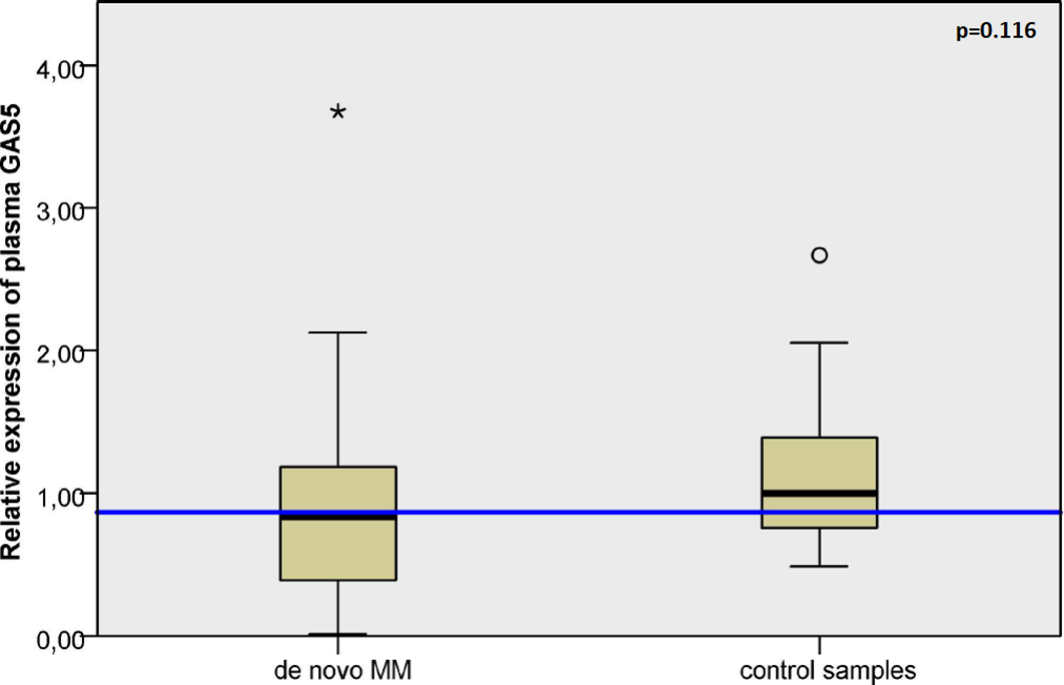
Comparison of circulating *GAS5* expression levels. Box-plot chart of relative *GAS5* expression level in the plasma of *de novo* MM patients (n=73) versus healthy controls (n=16).

The expression level of plasma *GAS5* was analyzed in the cohort of 73 *de novo* MM patients and compared to 16 control samples (**Figure 1**). The median expression level of plasma *GAS5* at diagnosis for MM patients was 0.837 (range 0.013 – 3.678). This level was marginally lower compared to that observed in control samples (median: 1.00; range: 0.490 – 2.668), however, this difference did not reach statistical significance (p = 0.116).

Using median expression level of plasma *GAS5* in MM cohort (0.837), we divided patients into two groups *GAS5* high (*GAS5*^high^) (n=37) and *GAS5* low (*GAS5*^low^) expressing patients (n=36). Analyzing the correlation between expression level of plasma *GAS5* and clinical characteristics of the patients, we have found that high expression *GAS5* was more often found in patients with high-risk cytogenetic abnormalities (double hit) (p=0.014) (**Table 1**). We haven’t found significant correlation with other clinical features, laboratory and FISH analyses (del17, t (4, 14), t (4, 16) and amp1q) and other prognostic parameters (**Table 1**).

### 4.3 Prognostic significance of plasma *GAS5* expression level

**Figure 2:**
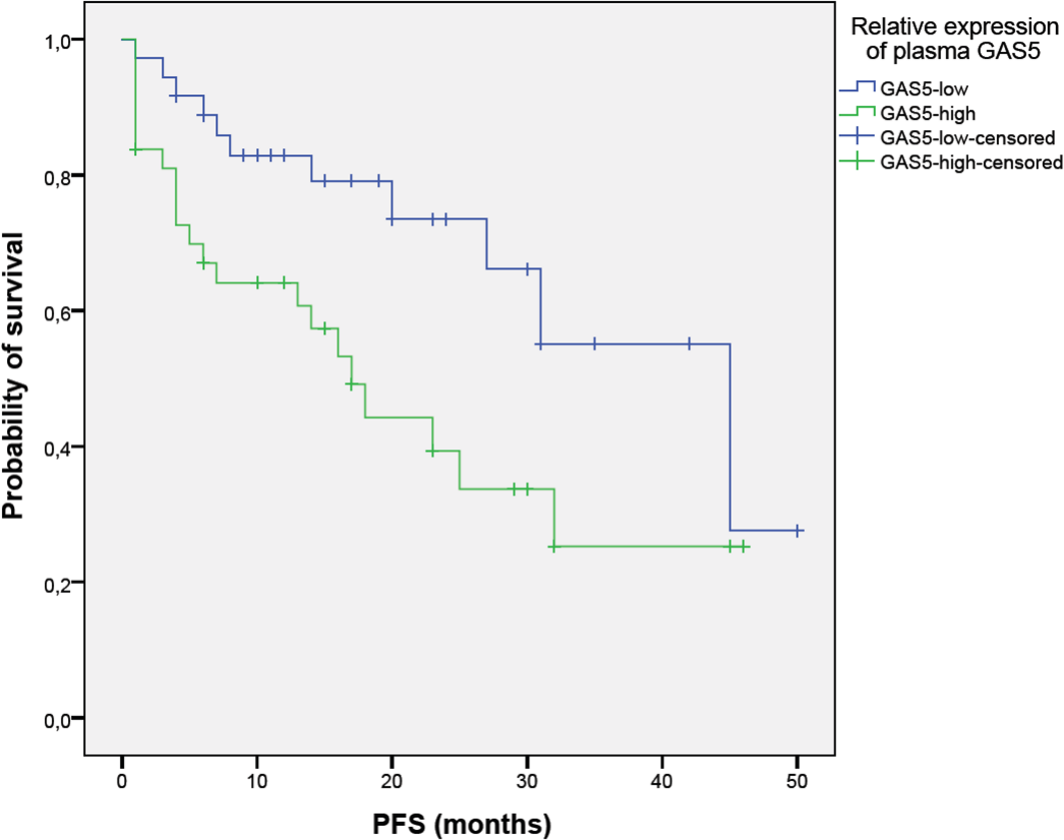
Impact of GAS5 expression on progression-free survival (PFS) in multiple myeloma. Kaplan-Meier analysis of PFS between GAS5^low^ (n=36; 17 censored) and GAS5^high^ (n=37; 12 censored) patients (45 vs. 17 months, log-rank=5.512, p=0.019). Groups were dichotomized based on the median GAS5 expression level.

**Figure 3:**
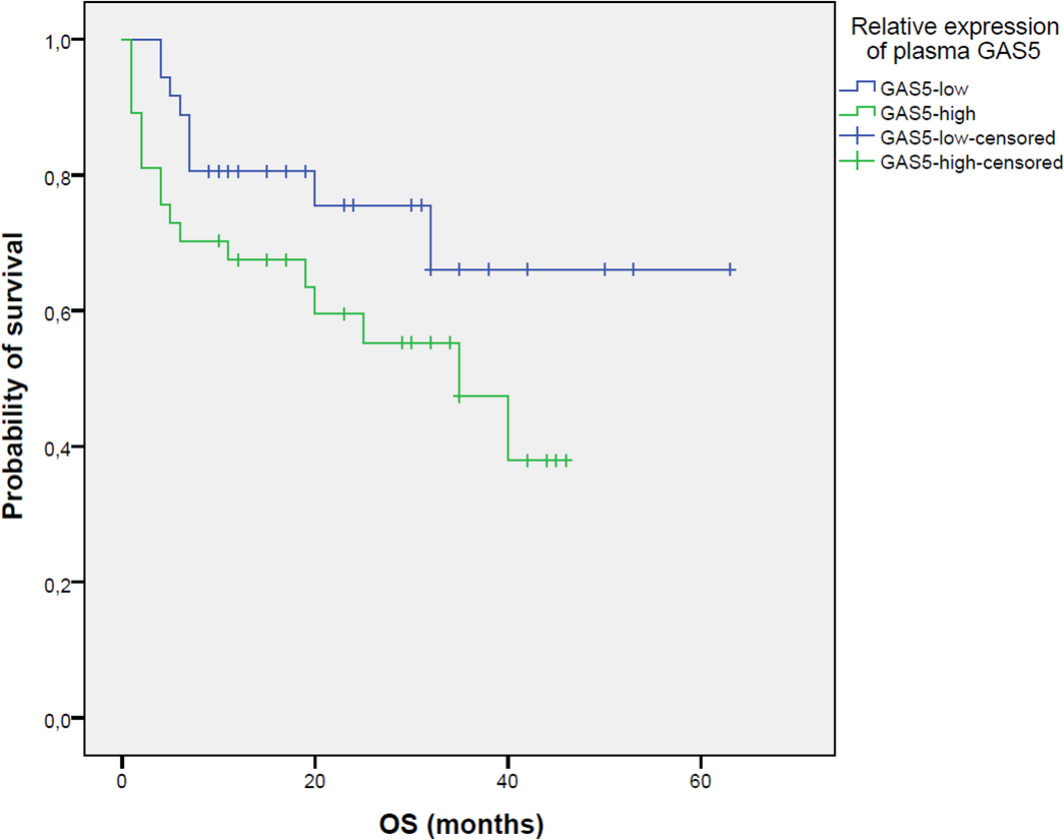
Kaplan–Meier analysis of overall survival (OS) between GAS5^low^ (n=36; 18 censored) and GAS5^high^ (n=37; 14 censored) patients (median not reached vs. 32 months, log-rank=3.127, p=0.077). Groups were dichotomized based on the median GAS5 expression level.

Using survival analysis, we tested the prognostic significance of plasma *GAS5* expression on PFS and OS. Patients with a high *GAS5* expression had a significantly shorter time to progression compared to *GAS5* low patients (17 vs. 45 months; log-rank=5.512, p=0.019) (**Figure 2**). Although *GAS5* high patients had inferior OS compared to *GAS5* low patients (not reached median vs. 32 months), the difference was not statistically significant (log-rank=3.127, p=0.077) (**Figure 3**).

In order to identify factors with the significant effect on PFS and OS we performed univariate and multivariate logistic regression analysis. Univariate analysis with OS as the dependent variable, demonstrated that age, R2-ISS score, and the occurrence of relapse or disease progression were significantly associated with OS. The multivariate regression model was adjusted for variables exhibiting a p<0.1 threshold in the univariate analysis. Advanced age (p=0.015; HR 1.058), a higher R2-ISS score (p=0.017; HR 2.774), and the occurrence of disease relapse and/or progression (p=0.041; HR 3.310) were identified as independent predictors of poorer OS (**Table 2**).

**Table 2:** Univariate and multivariate analysis with OS as dependent variable.

Clinopathological charcteristics	Univariate analyses		Multivariate analyses	
	p	HR (CI 95%)	P	HR (CI 95%)
Plasma *GAS5* expression	0.058	1.804 (0.981-3.316)	0.356	1.455 (0.656-3.225)
Sex (F/M)	0.148	1.796 (0.812-3.973)	–	
Age	0.008	1.058 (1.015-1.104)	0.015	1.058 (1.011-1.108)
Leukocytes	0.456	0.953 (0.839-1.082)	–	
Hemoglobin	0.339	0.991 (0.972-1.010)	–	
Platelets	0.576	1.001 (0.997-1.006)	–	
Creatinine	0.747	1.000 (0.998-1.002)	–	
Ca	0.214	0.507 (0.174-1.798)	–	
Immunoglobulin subtype	0.818	1.060 (0.643-1.748)	–	
Bone lesions	0.177	0.585 (0.268-1.275)	–	
Clinical stage (Durie-Salmon)	0.604	0.845 (0.448-1.596)	–	
ISS	0.252	1.436 (0.773-2.667)	–	
R-ISS	0.244	1.500 (0.758-2.969)	–	
R2-ISS	0.047	1.880 (1.009-3.506)	0.017	2.774 (1.196-6.431)
Treatment response (CR vs. VGPR, PR)	0.215	3.711 (0.462-29.472)	–	
Relapse/progression	0.003	5.244 (1.740-15.805)	0.041	3.310 (1.050-10.433)

**Figure 4:**
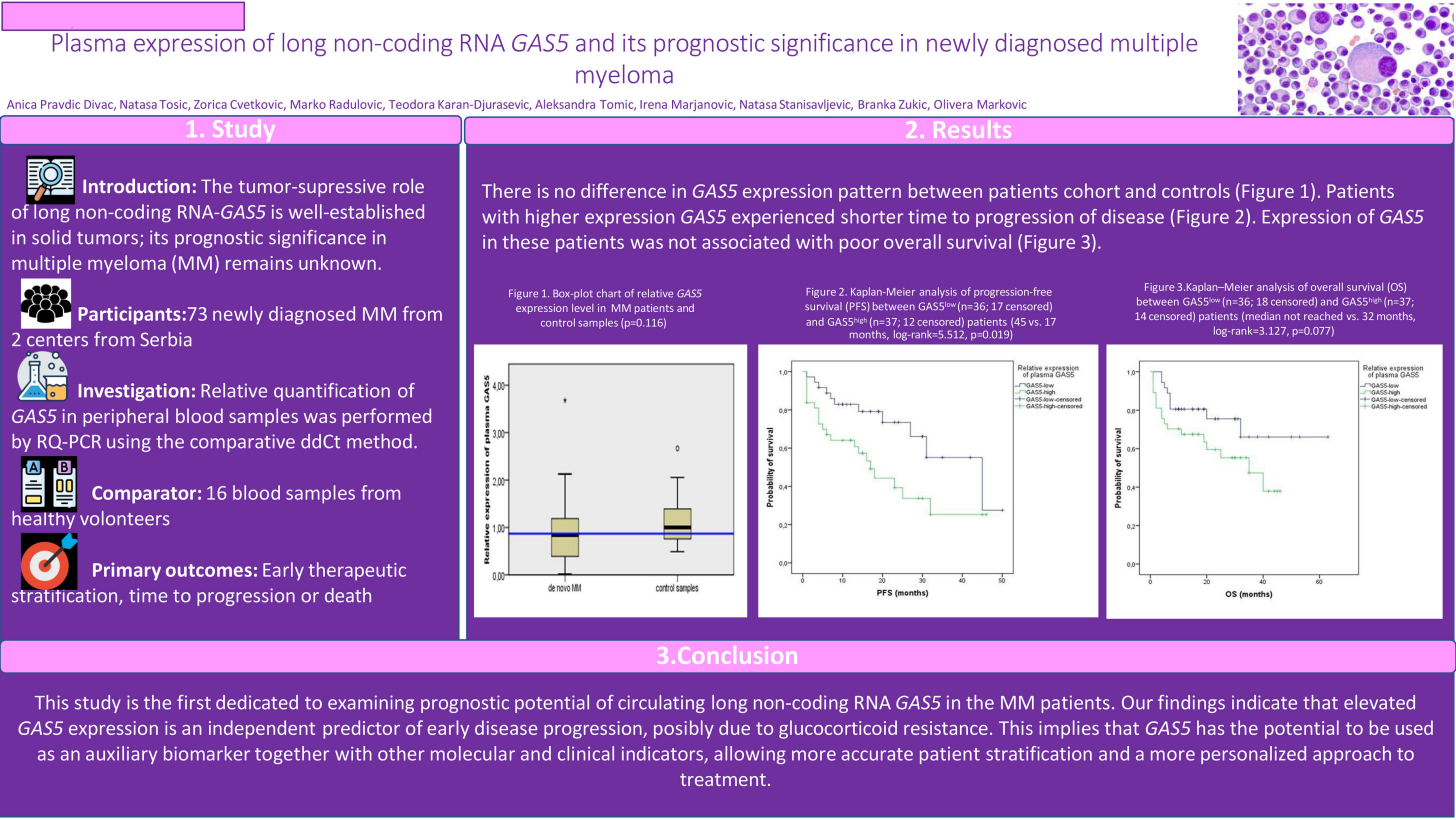
Graphical abstract of overall impact and significance of the study.

In the univariate analysis, where PFS was the dependent variable, *GAS5* expression (p=0.007; HR 2.139) and age (p=0.009; HR 1.055) emerged as statistically significant factors. This finding was confirmed in the multivariate analysis, which established that elevated plasma *GAS5* expression (p=0.023; HR 1.918) and advanced age (p=0.023; HR 1.046) are independent predictors of shorter time to progression (**Table 3**). The graphical abstract is shown in **Figure 4**.

**Table 3:** Univariate and multivariate analyses with PFS as dependent variable.

Clinicopathological characteristics	Univariate analyses		Multivariate analyses	
	p	HR(CI 95%)	P	HR(CI 95%)
Plasma *GAS5* expression	0.007	2.139 (1.228-3.729)	0.023	1.918 (1.094-3.361)
Sex	0.719	1.137 (0.566-2.285)	–	
Age	0.009	1.055 (1.014-1.098)	0.023	1.046 (1.006-1.087)
Leukocytes	0.433	0.954 (0.848-1.073)	–	
Hemoglobin	0.342	0.992 (0.975-1.009)	–	
Platelets	0.122	0.996 (0.992-1.001)	–	
Creatinine	0.256	1.001 (0.999-1.002)	–	
Ca	0.180	0.508 (0.188-1.368)	–	
Immunoglobulin subtype	0.488	0.843 (0.520-1.366)	–	
Bone lesions	0.492	0.767 (0.360-1.635)	–	
Clinical stage (Durie-Salmon)	0.908	0.962 (0.496-1.863)	–	
ISS	0.307	1.325 (0.772-2.273)	–	
R-ISS	0.196	1.495 (0.813-2.752)	–	
R2-ISS	0.254	1.358 (0.803-2.297)	–	
Treatment response (CR vs. VGPR, PR)	0.695	1.260 (0.397-3.995)	–	

The potential influence of patients’ clinical status on *GAS5* expression was evaluated, showing no significant differences between patients with and without comorbidities (p = 0.666). Furthermore, Spearman’s rank correlation confirmed the absence of a significant association between the number of comorbidities and GAS5 levels (rho = -0.525, p = 0.285) (data no shown).

## 5 Discussion

Over the past decade, many lncRNAs were extensively investigated in MM biology. LncRNAs have been shown as key regulators of proliferation, invasion and therapeutic resistance in MM (8, 11). Moreover, the evidence indicates that some lncRNAs may have prognostic significance, for example, overexpression of *MALAT1* is correlated with a shorter time to progression in MM patients (48). While the tumor-suppressive role of lncRA *GAS5* in various solid cancers is well-established, its prognostic significance in MM is still unknown. To the best of our knowledge, only two studies have been focused on investigating *GAS5* expression in MM and the results were contradictory. Our study addresses these discrepancies by utilizing circulating plasma samples, which offer a minimally invasive alternative that supports repeated sampling over time. In the context of multiple myeloma, this liquid biopsy approach is particularly advantageous as it overcomes the limitations of spatial heterogeneity inherent in bone marrow aspirates, ensuring that the detected markers reflect the global rather than localized bone marrow environment. Therefore, we analyzed the expression pattern of circulating plasma *GAS5* in *de novo* MM patients and examined its clinical significance.

Our study offers several distinct advantages and novel insights compared to existing literature. While previous research primarily focused on differential *GAS5* expression between patients and healthy controls, or broad survival outcomes, our analysis establishes a prognostic correlation between *GAS5* levels and PFS. Furthermore, we demonstrated for the first time that elevated *GAS5* expression is significantly associated with double-hit myeloma, a high-risk subgroup. Also, while Li and colleagues (38) examined transplant-eligible patients, our study focuses on transplant-ineligible individuals. This demographic represents a ‘real-world’ patient population. By integrating *GAS5* data with laboratory characteristics, demographic features, and established prognostic scores, our findings provide a more clinically applicable predictive model than previously reported.

We found that the *GAS5* expression level in MM was slightly lower than expression in healthy controls. This finding is in contrast to the report from Isin et al. where the expression level of plasma *GAS5* in MM patients was significantly lower compared to controls (37). But, in the report by Li et al. serum *GAS5* expression in MM patients was significantly higher than control samples (38). Expression analysis of *GAS5* in some hematological malignancies like acute myeloid leukemia, diffuse large B−cell lymphoma and B lymphocytic leukemia showed a significant decrease in the expression of this lncRNA (33, 34, 49). The lack of significant difference in *GAS5* expression between patients and healthy controls suggests that *GAS5* may not serve as a primary diagnostic biomarker in this study. Instead, its significance lies in its prognostic significance within the MM patients, where its upregulation may reflect an aggressive tumor biology or resistance to treatment, rather than in tumorigenesis. Downregulation of *GAS5* expression is consistent with its presumed tumor-suppressor function in pathogenesis of tumor (50). However, there are types of cancer where *GAS5* expression is similar to the control samples, or it is even significantly increased (29, 32, 35, 51, 52). For instance, significant upregulation of *GAS5* was found in plasma of mesothelioma patients, and also in the plasma of patients with myelofibrosis (31, 35). Based on these findings, it can be assumed that in some malignancies *GAS5* may also act as an oncogene. For example, overexpression of *GAS5* in patients with hepatocellular carcinoma, mesothelioma, cholangiocarcinoma and myelofibrosis were associated with poor prognosis (29, 31, 32, 35). The heterogeneous expression patterns of *GAS5* across various malignancies suggests its tissue-specific role (53). The various effects of *GAS5* in different malignancies and even in different patients with the identical type of cancer, could be a consequence of a heterogenous tumor microenvironment as well as the presence of numerous cytogenetic aberration that can regulate the effect of *GAS5* in tumor cells.

Furthermore, we analyzed prognostic significance of *GAS5* in MM and found that group with higher *GAS5* expression associated with poor PFS. Our finding may support the role of *GAS5* in glucocorticoids resistance in MM. *In vitro* studies confirmed the *GAS5* molecular interaction with GR. *GAS5* acts as a molecular decoy and inhibits the most important effect of glucocorticoids in the nucleus, i.e., it leads to the inhibition of tumor cell apoptosis (17). Also, Lucafo and colleagues showed that higher *GAS5* expression in peripheral blood mononuclear cells (PBMC) from healthy donors was associated with a poorer response to methylprednisolone compared to PBMC with lower *GAS5* expression (54). This effect of *GAS5* on GR may explain the worse PFS in MM patients with elevated *GAS5* expression despite the use of standard therapy. Similar results were shown in studies that confirmed elevated expression of *GAS5* in steroid-resistant asthma (55), as well as in patients with poor response to therapy and early relapse of childhood acute lymphoblastic leukemia (36). Building on this, we specifically addressed whether the presence of comorbidities or treatment adjustments could have influenced our results. In our cohort, no significant associations were found between GAS5 levels and baseline health status, indicating that its predictive value is not confounded by the patients’ general clinical condition. This independence suggests that elevated GAS5 reflects an intrinsic mechanism of therapeutic resistance rather than being a mere byproduct of comorbidity-related treatment modifications. Consequently, our study suggests that plasma GAS5 may serve as a robust, independent biomarker for predicting earlier disease progression in MM.

Still, in addition to the results obtained related to PFS, *GAS5* expression does not maintain the same level of significance for OS. This distinction is clinically relevant and biologically plausible. LncRNA *GAS5* as biomarker appears more directly to progression of the disease and the time of first recurrence. In contrast, OS is a complex endpoint influenced by multiple confounding variables, most notably the impact of subsequent lines of therapy following relapse. In the modern treatment landscape of multiple myeloma, effective salvage therapies and intensification strategies can successfully extend survival post-progression and effectively dissociating initial molecular prognostic indicators from the OS.

Moreover, in multivariate analysis older age, higher R2-ISS scores, and the occurrence of relapse or progression were independent predictors of poor survival in our cohort. Advanced age represents a strong predictor for inferior OS and PFS in our group. This finding is consistent with several previous studies (4, 56–58). Limited treatment choices and reduced biological reserve are potential factors contributing to poor prognosis in this population. In this population treatment is challenging because of limitations imposed by increased risk of side effects and toxicity. However, the appearance of glucocorticoid resistance represents one of the main barriers in achieving long-term remission, especially in elderly patients where therapeutic options are often limited (59). Additional effort is needed to design better comprehensive frailty assessments and improve available therapeutic options for this population.

In one of the attempts to better stratify MM patients, D’Agostino and colleagues defined a revised second International Prognostic Scoring System (R2-ISS). The aim of the study was better stratification of patients within the R-ISS 2 group. This model integrates specific high-risk cytogenetic aberrations—such as del(17p) and amp (1q) with laboratory results (lactate dehydrogenase value) ​​and older versions of prognostic score (ISS stages 2 or 3) (41). Consistent with our research, a higher R2-ISS score correlates with significantly shorter OS. It is important to point out that the original study included younger patients who were candidates for ASCT, while our cohort consists mostly of older patients who are transplant-ineligible. These results indicate that the R2-ISS demonstrates strong predictive performance in patients from real-world clinical practice.

Despite the observed data, this study has several limitations. Firstly, the study has a small cohort of patients from two tertiary centers. In addition, the majority included patients who were not eligible for ASCT. It is necessary to conduct a study on a larger number of patients in several centers in order to obtain a conclusion that can be applied to all cohorts of MM patients. Secondly, samples in study were taken at the time of diagnosis. In future, it is essential to have longitudinal monitoring, taking samples during treatment, also at the moment of achieving remission or occurrence of relapse/progression. Although the recruitment period (July 2020–December 2024) allowed for a maximum follow-up of over five years for the earliest participants, the median follow-up for the entire cohort was 29 months. This indicates that while the data is mature enough to establish a significant difference in PFS—with the *GAS5*^low^ group reaching a median PFS of 45 months compared to 17 months in the *GAS5*^high^ group—the results for more recently enrolled patients remain early. Consequently, the long-term impact on OS may require further observation, as OS is often influenced by subsequent lines of therapy following initial progression. Future studies with extended follow-up are warranted to confirm the long-term prognostic stability of plasma *GAS5* in this patient population. Our results could represent a basis for future research in order to develop personalized treatment in MM patients.

## 6 Conclusion

This study is the first dedicated to examining prognostic potential of circulating lncRNA *GAS5* in the MM patients. Our findings indicate that elevated *GAS5* expression is an independent predictor of early disease progression, possibly through modification of corticosteroid response. This implies that *GAS5* has the potential to be used as an auxiliary biomarker together with other molecular and clinical indicators, allowing more accurate patient stratification and a more personalized approach to treatment. However, only further studies enrolling larger number of MM patients could elucidate if circulating *GAS5* expression is a suitable biomarker for clinical utilisation.

## Data Availability

The original contributions presented in the study are included in the article/supplementary material. Further inquiries can be directed to the corresponding author.
